# Calcitonin gene-related peptide and pain: a systematic review

**DOI:** 10.1186/s10194-017-0741-2

**Published:** 2017-03-16

**Authors:** Wendy Sophie Schou, Sait Ashina, Faisal Mohammad Amin, Peter J. Goadsby, Messoud Ashina

**Affiliations:** 1 Danish Headache Center, Department of Neurology, Rigshospitalet Glostrup, University of Copenhagen, Copenhagen, Denmark; 2 Department of Neurology, NYU Lutheran Headache Center, New York University School of Medicine, NYU Langone Medical Center, New York, NY USA; 3 Basic & Clinical Neuroscience, and NIHR-Wellcome Trust King’s Clinical Research Facility, King’s College London, London, UK

## Abstract

**Background:**

Calcitonin gene-related peptide (CGRP) is widely distributed in nociceptive pathways in human peripheral and central nervous system and its receptors are also expressed in pain pathways. CGRP is involved in migraine pathophysiology but its role in non-headache pain has not been clarified.

**Methods:**

We performed a systematic literature search on PubMed, Embase and ClinicalTrials.gov for articles on CGRP and non-headache pain covering human studies including experimental studies and randomized clinical trials.

**Results:**

The literature search identified 375 citations of which 50 contained relevant original data. An association between measured CGRP levels and somatic, visceral, neuropathic and inflammatory pain was found. In 13 out of 20 studies in somatic pain conditions, CGRP levels had a positive correlation with pain. Increased CGRP levels were reported in plasma, synovial and cerebrospinal fluid in subjects with musculoskeletal pain. A randomized clinical trial on monoclonal antibody, which selectively binds to and inhibits the activity of CGRP (galcanezumab) in patients with osteoarthritis knee pain, failed to demonstrate improvement of pain compared with placebo. No studies to date have investigated the efficacy of monoclonal antibodies against CGRP receptor in non-headache pain conditions.

**Conclusion:**

The present review revealed the association between measured CGRP levels and somatic, visceral, neuropathic and inflammatory pain. These data suggest that CGRP may act as a neuromodulator in non-headache pain conditions. However, more studies are needed to fully understand the role of CGRP in nociceptive processing and therapy of chronic pain.

**Electronic supplementary material:**

The online version of this article (doi:10.1186/s10194-017-0741-2) contains supplementary material, which is available to authorized users.

## Background

The mechanism of nociception is complex involving the detection of a noxious event by nociceptors, and signal processing in the peripheral and central nervous system (CNS). Recent studies have identified specific substances and receptors with potential roles in nociception that provide therapeutic targets, including substance P, CGRP, glutamate, serotonin, TrkA receptor, vanilloid receptor and NMDA receptor [1, 2]. Chronic pain resulting from disease or injury is a major public health problem and a common complaint in general population with a lifetime prevalence ranging from 12 to 30% [3] and an enormous impact and burden on society and individuals [4]. Despite tremendous scientific effort over the past years, current pain management treatment remains suboptimal [5]. There is an unmet and urgent need for new effective therapeutic options for the management of chronic pain. Migraine manifests as pain with associated sensory disturbances and is considered as a chronic condition with episodic manifestations [6]. The role of CGRP in migraine pathophysiology has gained considerable interest in recent years [7, 8]. This led to the development of small molecule CGRP receptor antagonists for acute and preventive treatment of migraine [9, 10] and monoclonal antibodies against CGRP mechanisms for migraine prevention [11, 12].

CGRP is a 37-amino-acid neuropeptide identified in 1982 [13]. It belongs to a family of peptides including adrenomedullin, amylin and calcitonin with diverse biological functions in the periphery and in the central nervous system [14, 15]. To what extent CGRP is involved in non-headache pain conditions is not fully clarified and whether CGRP antagonism may represent a useful therapeutic approach for the treatment of chronic pain is unknown.

The aim of this systematic review was to assess the role of CGRP in non-headache pain in humans. In addition we discussed the potential role of anti-CGRP agents in the management of chronic pain.

## Methods

### Literature search

We performed a systematic literature search identifying articles reporting original data on CGRP and non-headache pain. We concluded the literature search on Pubmed Embase and ClinicalTrials.gov on May 2016. We used the following search terms: CGRP and pain. In addition, we specified our search criteria in ClinicalTrials.gov to currently available monoclonal antibodies against CGRP (LY2951742, ALD-403, PF-04427429, LBR-101/TEV-48125) or its receptor (AMG334), and CGRP receptor antagonists (BIBN4096BS, MK-0974, MK-3207, MK-1602, MK-8825, BMS-694153, BMS-927711, BMS-742413, BI 44370 TA) [16].

Only human studies published in English language were included. Review papers editorials and other articles without original data were excluded. We also considered articles from the reference list of studies that were found to be relevant as well as literature that was known to be relevant by the authors.

### Data extraction

The authors (WSS) examined the abstracts found in the literature search. Whenever the title or abstract suggested that relevant data could be part of the publication the entire article was read and discussed with the other co-authors. Studies in which patients had unclear pain history or articles without relevant data on CGRP were not included in the review.

## Results

**Fig. 1:**
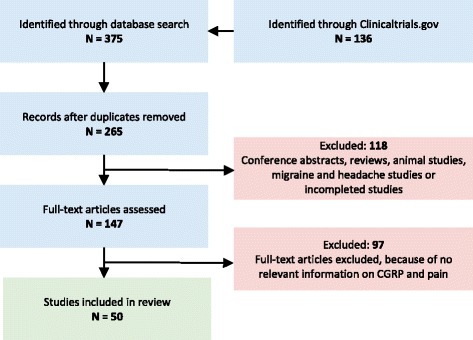
Flow chart of the study

**Fig. 2:**
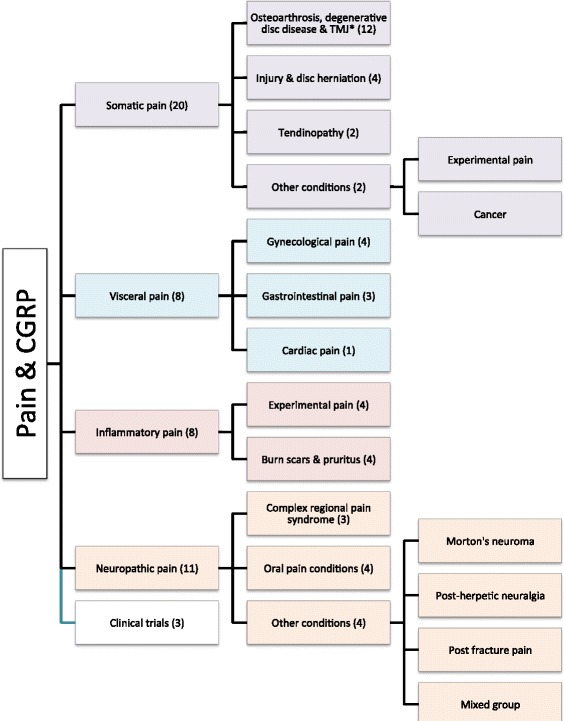
Overview of the studies in non-headache pain included in each category in the present review

Our Pubmed Embase and ClinicalTrials search strategy identified 375 hits of which 50 studies were included in the final review (Fig. 1). After excluding 110 as duplicates, another 118 hits were excluded because these were abstracts, reviews, animal, migraine, headache studies, and incomplete studies. Subsequently, we excluded 97 studies that did not describe pain history of the patients, had no relevant data on CGRP, or had unclear methodology. In total, 50 studies were included in the final review (Fig. 1). The identified studies were further divided into five categories: 1) somatic pain, 2) visceral pain, 3) inflammatory pain, 4) neuropathic pain, and 5) clinical trials (Fig. 2).

### Somatic pain

We found a total of 20 studies on the role of CGRP in somatic pain (Table 1). Using different methodological approaches and CGRP sample sources 13 studies showed higher levels of CGRP compared to controls. Eight studies directly tested for a possible correlation between pain intensity and CGRP levels. In chronic knee pain due to osteoarthrosis elevated CGRP levels were detected in serum and synovial fluid in patients compared with controls. Serum CGRP levels were positively correlated with pain intensity [17]. Chronic low back patients due to osteoarthrosis showed decreased blood CGRP levels four months after successful auricular point acupressure pain treatment compared to baseline. No decrease was found in patients who received sham treatment [18]. In addition, studies using immunofluorescence of skin biopsies reported decreased CGRP after acupuncture treatment of osteoarthrosis patients [19]. Immunohistochemistry analysis of synovial tissue from fossa acetabuli showed increased CGRP levels in patients compared to controls [20, 21]. One study reported higher levels of CGRP in hip synovium from osteoarthritis patients compared with femoral neck patients [22]. Moreover, one study [23] revealed higher levels of CGRP in synovial tissue from temporomandibular joint (TMJ) pain patients compared with controls. This study also reported positive correlation between pain and CGRP levels [23]. Biopsies from knee joint ligaments showed no difference in CGRP nerve density between patients and non-arthrosis patients [24]. In patients with osteoarthrosis CGRP concentration in cerebrospinal fluid was decreased compared to controls [25].

**Table 1:** Studies on the role of CGRP in somatic pain

Study	Objectives	Reported pain as part of phenotype	Method and sample size	Source of CGRP	Results	Duration of the investigated condition	Correlation between CGRP level and pain
Alpar, 2002 [1]	Determine plasma CGRP in patients with whiplash injury who were treated by carpal tunnel decompression	Chronic shoulder and neck pain due to whiplash injury	38 patients and 11 controls. Enzyme-immunoassay kit was used to measure the plasma CGRP	Blood (plasma)	Mean plasma levels was higher in patients, 400 ng/l, than in controls, 85 ng/l. Plasma levels were reduced, 65 ng/l, after carpal tunnel decompression	NR	Reduced plasma CGRP after the operation correlated to the pain reduction
Bjur, 2005 [2]	Investigate innervation patterns of Achilles tendon in tendinosis tendon, and normal tendon	Chronic pain in tendinosis	Tissue samples from 21 patients and 9 controls	Tissue biopsies (Achilles tendon)	Inconclusive. CGRP was found in both patients and controls. The amount of CGRP-fibers was not quantified	Mean 19 months	NR
Brown, 1997 [3]	Determine density of CGRP containing sensory nerve fibers in vertebral endplate in patients with degenerative disc disease	Severe back pain with or without sciatica in degenerative disc disease	Tissue from the intervertebral discs from 15 patients undergoing anterior lumbar discectomy and 7 healthy post-mortem controls	Tissue biopsies (intervertebral discs)	Marked increase in CGRP-containing sensory nerve fibers compared with controls	NR	NR
Carlsson, 2006 [4]	Evaluate possible effects of acupuncture on sensory nerve fibers in human skin	Cervicobrachial pain, cervicocranial pain, hip pain and finger pain from arthrosis	Punch skin biopsies taken from 6 patients one week before acupuncture and 3–6 days after the 10th treatment	Tissue biopsies (skin)	The mean number of CGRP-IR nerve fibers were reduced after treatment	4 months - >10 years	NR
Danielson, 2008 [5]	Investigate CGRP prevalence in patients with tendinitis surgery.	Chronic painful patellar tendinosis	Patellar tendon biopsy in 7 patients	Tissue biopsies (patellar tendon)	CGRP rarely detected at perivascular sites	Chronic pain	NR
Dong, 2015 [6]	Examine CGRP concentrations in patients with primary knee OA and controls	Chronic knee pain from OA	Serum CGRP concentrations in OA patients (*n =* 65) and controls (*n =* 21).	Blood (serum)	CGRP levels were higher in patients, 2.43 ng/mL, than in controls, 1.95 ng/mL	NR	CGRP concentrations in serum were correlated with pain intensity
Ikeuchi, 2012 [7]	Determine sensory innervation of posterior cruciate ligament (PCL) in patients with OA	Chronic knee pain from OA	PCL samples from 10 patients and 5 pain-free controls with anterior cruciate ligament (ACL) rupture	Tissue biopsies (joint ligament)	No difference between patients and controls	NR	NR
Jonhagen, 2006 [8]	Determine CGRP in human skeletal muscle at rest and after painful eccentric exercise	Experimental muscle pain after eccentric exercise	Microdialysis catheter inserted in quadriceps muscle in 8 healthy volunteers. Samples taken before and after exercise.	Blood (plasma)	CGRP levels were higher 2 days after exercise, 5.4 fmol/ml, than directly after exercise, 4.85 fmol/ml	VAS-score was assessed on the entry day (VAS = 0), day 1 (VAS = 1) and day 3 (VAS = 2) after the exercise	CGRP concentrations was positively correlated with pain intensity (VAS)
Larsson, 1991 [9]	Investigate CGRP-levels from patients with rheumatoid arthritis and patients with meniscal/cruciate ligament injuries	Acute knee pain in meniscal/cruciate ligament injuries	Synovial fluid from the knee joint of 18 patients and 13 pain-free controls with ligament injuries	Synovial fluid (knee joint)	Increased CGRP levels in patients compared to controls.	4-27 years	NR
Lin, 2015 [10]	Investigate associations between plasma CGRP-levels and clinical outcome from APA, in patients with osteoarthritis and spinal stenosis	Chronic lower back pain (CLBP) in patients with osteoarthritis and spinal stenosis	Blood samples from 32 patients (APA-group) and 29 controls (sham APA-group). Samples were taken at baseline and 4 weeks later. VAS-score before treatment was 4.	Blood (plasma)	Patients showed a decrease in CGRP levels after treatment. No decrease in the control group	At least 3 months	56% of the patient group reported a reduction in pain, whereas only 9% controls reported a reduction
Lindh, 1999 [11]	Determine CGRP-LI levels in CSF in patients with chronic pain	Osteoarthritis, herniated lumbar disc and hip fracture pain	Sample: CSF Subjects: 35 patients (14 had knee or hip pain, 11 had rhizopathic pain due to herniated lumbar pain, 10 had pain from hip fracture and 12 healthy controls. Pain assessment: VAS	CSF	Decreased CGRP-LI levels were observed in patients compared to healthy controls	Osteoarthritis patients: >6 months. Rhizopathic pain: At least 1 month (1–13). Hip fracture pain: Up to 48 h	No correlation between CGRP-LI levels and VAS-values could be observed for any of the subjects participating in the study
Onuoha, 1999 [12]	Investigate CGRP levels in patients with soft tissue injury	Acute muscle and ligament pain due to injury	Plasma CGRP-concentrations in 17 patients and 15 healthy controls	Blood (Plasma)	CGRP-levels were significantly higher in patients than controls	Up to 24 h	NR
Ozawa, 2006 [13]	To determine sensory fibers innervating human degenerated lumbar intervertebral discs	Discogenic low back pain	Lumbar intervertebral disc was harvested from 8 patients, and immunostained for CGRP	Tissue biopsies (intervertebral disc)	CGRP-IR nerve fibers were observed in 6 out of 8 patients	NR	NR
Samuelsson, 1993 [14]	Determine CSF CGRP levels in cancer patients	Cancer pain	CSF from 10 patients compared with 10 controls	CSF	No difference in CGRP-levels between patients and controls	NR	No difference between patients with pain and controls
Sasaki, 2013 [15]	Investigate innervation patterns of ECRB in patients with recalcitrant tennis elbow	Lateral epicondylitis	Tissue biopsies from 8 patients and 2 controls. The control group suffered from osteochondritis	Tissue biopsies (lateral epicondyle)	A decrease in the immunorectivity of CGRP compared to controls	Mean duration 23 months	The innervation pattern did not appear to be correlated with VAS-score
Sato, 2004 [16]	Elucidate expression of CGRP in temporomandibular joint (TMJ) from patients with internal derangement	TMJ pain	Synovial fluid from 48 patients and 7 controls, who had pain-free habitual dislocation	Synovial fluid (TMJ)	Increased CGRP in patients compared to controls	Mean duration 6 months	Positive correlation between the extent score of CGRP-levels and joint pain
Saxler, 2007 [17]	Determine presence of CGRP-immunopositive nerve fibers in patients with OA	Hip pain from OA	Soft tissue biopsies from fossa acetabuli in 3 patients and 6 pain-free controls. 3 controls had a failed THA and 3 controls had femoral neck fractures	Tissue biopsies (fossa acetabuli)	Increased CGRP-LI in patients compared to controls.	NR	Positive correlation between CGRP and pain
Takeshita, 2012 [18]	Clarify sensory innervation and inflammatory cytokines in OA patients	Severe hip pain from OA	Synovium from 50 patients and 12 controls with femoral neck fracture	Synovial fluid (hip)	CGRP-IR sensory nerve fibers were observed in 54% of the patients and 0% in controls	NR	NR
Takeuchi, 2007 [19]	Determine CGRP’s role in patients with lumbar disc herniation, before and after lumbar discectomy	Sciatic pain/lumbar disc herniation	Plasma CGRP was measured in 27 patient before and 3 weeks after lumbar discectomy	Blood (plasma)	Plasma CGRP-levels were reduced after lumbar discectomy	3 weeks	Reduced plasma CGRP after the operation correlated to lower VAS-levels
Wang, 2015 [20]	Explore mechanisms of possible involvement and regulation of CGRP in pathological and inflammatory processes of arthritis in patients with developmental dysplasia of the hip (DDH)	Hip pain from OA	Synovial tissue samples from 67 patients: 35 with moderate DDH and 32 patients with severe DDH. 15 controls with traumatic femoral fracture	Synovial tissue (fossa acetabuli)	Increased CGRP in synovium fluid from patients in the severe DDH group compared to the moderate DDH group and controls	NR	The highest amount of CGRP correlated with the highest VAS

*APA* Auricular point acupressure, *CGRP* Calcitonin gene-related peptide, *CGRP-LI* Calcitonin gene-related peptide-like immunoreactivity, *CLBP* Chronic low back pain, *CRPS* Complex regional pain syndrome, *CSF* Cerebrospinal fluid, *ECRB* Extensor carpi radialis brevis, *KL grades* Kellgreen and Lawrence classification, used to assess the severity of OA, *NR* not reported, OA Osteoarthritis, *PHN* Postherpetic neuralgia, *THA* Total hip arthroplasties

In patients suffering from chronic pain due to degenerative disc disease disc biopsies showed increased CGRP compared to post-mortem control discs [26]. Biopsies from intervertebral discs in patients with low back pain contained CGRP-IR nerve fibers [27].

Patients suffering from shoulder and neck pain due to whiplash injury were found to have higher blood CGRP levels compared to controls [28]. Another study of patients with disc herniation pain reported increased blood levels of CGRP which were normalized after discectomy [29]. Blood CGRP levels were also elevated in patients with soft tissue injury (i.e. muscle or ligament pain) compared with controls [30]. Furthermore, one radioimmunoassay study of knee synovial fluid from patients with meniscal or ligament injury revealed higher CGRP levels compared to controls [31].

Immunohistochemistry analysis of biopsies of Achilles tendons from patients with chronic painful tendinosis showed no changes in CGRP levels in patients compared to controls [32] while another study in patients with patellar tendinosis found the presence of CGRP, but had no control group [33]. One study reported decreased CGRP in the extensor carpi radialis brevis tendon biopsy from patients with tennis elbow compared to patients with osteochondritis [34].

Samuelsson and colleagues [35] compared CGRP levels in cerebrospinal fluid from cancer patients with pain and found no difference between patients and non-pain control patients. A microdialysis study in the vastus lateralis of the quadriceps muscles before and during pain after eccentric exercise (repetitive muscle contractions while the muscle is lengthening under load) reported increased CGRP levels during pain compared with baseline [36].

### Visceral pain

Eight studies examined CGRP in different types of visceral pain conditions (Table 2).

**Table 2:** Studies on the role of CGRP in visceral pain

Study	Objectives	Reported pain as part of phenotype	Method and sample size	Source of CGRP	Results	Duration of the investigated condition	Correlation between CGRP level and pain
Arellano, 2011[1]	Investigate nerve growth factor role in development of pelvic pain in patients with endometriosis	Pain from endometriosis	Peritoneal fluids from 65 patients, 54 with pain, 11 without pain. 22 controls, where 12 reported pelvic pain	Peritoneal fluid	CGRP-neurite outgrowth was seen in patients	NR	The CGRP-neurite outgrowth did not correlate with pain symptoms
Büchler, 1992 [2]	Identify characteristics of peptidergic innervation in patients with chronic pancreatitis	Pain from chronic pancreatitis	Pancreatic tissue from 20 patients compared to 10 organ donors	Tissue biopsies (pancreatic tissue)	CGRP-immunostaining was intensified in patients	NR	NR
Mönnikes, 2005 [3]	Assess whether functional dyspepsia (FD) patients have altered mucosal CGRP concentrations	Pain from functional dyspepsia	Gastric mucosal biopsies from 13 patients and 18 controls. Biopsies were taken during gastric distention	Tissue biopsies (gastric mucosa)	No difference in CGRP-levels between patients and controls	The gastric distention took up to 80 min	A negative correlation between CGRP concentrations and pain was observed in patients. No such correlation was found in controls
Tokushige, 2006 [4]	Determine the nerve fibers in patients with peritoneal endometriosis	Pain from endometriosis	Peritoneal endometriotic tissue from 40 patients and 36 healthy controls. Also 9 specimens from endosalpingiosis lesions were prepared	Tissue biopsies (endometriotic tissue)	Increase of CGRP-nerve fibers in patients, compared to controls and endosalpingiosis lesions	NR	NR
Tokushige, 2007 [5]	Investigate types of nerve fibers in endometrium and myometrium in women with endometriosis	Pain from endometriosis	Tissue biopsies from 10 patients and 35 controls. All tissue biopsies were taken during hysterectomy	Tissue biopsies (endometriotic tissue)	Increased nerve fiber densities compared to controls	NR	NR
Tympanidis, 2003 [6]	Evaluate nerve fiber density and pattern in patients with vulvodynia	Pain from vulvodynia	Biopsies from the wall of the vulval vestibule from 12 patients and 8 controls	Tissue biopsies (vulval vestibule)	No difference in CGRP-immunostaining between patients and controls	NR	NR
Währborg, 1999 [7]	Clarify potential involvement of CGRP in anginal pain and myocardial ischemia in humans	Chest pain from angina and acute myocardial infarction	Plasma from 87 patients with AMI compared to 14 patients with severe angina pectoris	Blood (plasma)	No difference in CGRP-levels between patients with AMI and angina pectoris	At least 15 min	No correlation between CGRP-levels and pain
Yoshida, 2013 [8]	Estimate expression of CGRP in esophageal mucosa in nonerosive reflux disease (NERD) patients	Pain due to NERD	Biopsies from 24 patients, compared to 24 controls	Tissue biopsies (esophageal mucosa)	No difference in CGRP-levels between patients and controls	NR	NR

Immunofluorescence-based analysis of peritoneal fluid obtained during diagnostic laparoscopy in patients with endometriosis showed increased CGRP levels compared to peritoneal fluid from controls without endometriosis [37]. Visual analogue scale scores were registered in all patients but authors found no correlation between CGRP levels and severity of pain. Immunohistochemistry analyses of peritoneal endometriotic lesions and normal peritoneum from non-endometriotic women showed increased CGRP in affected tissue material [38]. Using the same technique, increased CGRP levels were found in endometrium and myometrium in women with, but not in those without endometriosis. Pain measurement data was not reported [39]. CGRP levels were also studied in patients with vulvodynia. Analysis of vulval vestibule tissue revealed no differences in CGRP levels between patients with vulvodynia and controls [40].

Gastric mucosal biopsies from patients with non-erosive reflux disease [41] and functional dyspepsia [42] were investigated with enzyme- and radioimmunoassay. None of the studies found differences in CGRP levels between patients and controls but a negative correlation between CGRP concentrations and pain scores was reported in the latter [42]. CGRP has also been investigated with immunohistochemistry in patients with alcohol-based painful chronic pancreatitis and increased CGRP levels in patients were reported compared with pancreatic tissue from organ donors [43].

Plasma CGRP levels were studied in patients with suspected or definite acute myocardial infarction at admission at a coronary care unit [44]. This study revealed no difference in CGRP levels between patients with and without acute myocardial infarction and no difference between patients with pain and those without pain.

### Inflammatory pain

Eight studies on CGRP and inflammatory pain conditions were identified (Table 3). ELISA of dermal microdialysate from volar forearm showed elevated blood CGRP levels in ten healthy volunteers with capsaicin-induced pain [45]. No CGRP release was detected via dermal microdialysate after electrical stimulation in the same area. Correlation between pain intensity or threshold and CGRP concentration was not tested [45]. In contrast another study found CGRP in the dialysate after histamine iontophoresis, but not after capsaicin application in the volar forearm [46]. One study performed immunohistochemistry of skin biopsies after intradermal capsaicin injection and reported complete loss of CGRP visualization 72 h after injection [47].

**Table 3:** Studies on the role of CGRP in inflammatory pain

Study	Objectives	Reported pain as part of phenotype	Method and sample size	Source of CGRP	Results	Duration of the investigated condition	Correlation between CGRP level and pain
Geber, 2007 [1]	Evaluate pain, hyperalgesia and neurosecretory function in pain models with CAP and ES	Experimental pain: CAP and ES	Samples from dermal microdialysis taken from 10 healthy volunteers. Patients rated pain levels after CAP and ES stimulation	Blood (plasma)	CGRP increase was measured after CAP, not after ES	2 h	NR
Hamed, 2011 [2]	Determine cutaneous innervation in burn patients with chronic pain	Chronic inflammatory skin pain	Skin biopsies from 12 patients and 33 controls suffering from unilateral injury, without pain	Tissue biopsies (skin)	Increase in CGRP density compared to controls	>24 months	CGRP-levels were higher in patients with pain compared to controls
Krämer, 2005 [3]	Explore effect of specific blockers of NEP (phosphoramidon) and ACE (captopril) on intensity of neurogenic inflammation	Experimental pain: ES	Samples from dermal microdialysis were taken from 8 healthy volunteers. Patients quantified pain sensation during electrical stimulation using VAS	Dermal microdialysis	CGRP release could be measured after phosphoramidon perfusion	1 h	CGRP release did not correlate to pain ratings during phosphoramidon infusion
Kwak, 2014 [4]	Evaluate CGRP’s effect on wound healing process in hypertrophic scar formation	Inflammatory pain in scars	Skin biopsies from 43 patients. Biopsies were taken from scars, and also from a normal skin area	Tissue biopsies (skin)	Increased CGRP-levels in scars compared to matched unburned skin	>12 months	Increased CGRP-levels in painful scar areas compared to normal skin
Onuoha, 2001 [5]	Examine plasma CGRP levels in patients with burns	Inflammatory pain from burn	Plasma was obtained from 13 patients immediately on hospital admission and 24 h after admission. 13 volunteers served as controls	Blood (plasma)	CGRP levels were higher on admission, 4.9 pmol/L and after 24 h, 7.3 pmol/L, than in controls, 1.9 pmol/L	NR	NR
Salomon, 2008 [6]	Evaluate CGRP-levels in AD patients during exacerbation and disease remission	Pruritus due to AD	Plasma from 49 patients and 32 healthy controls	Blood (plasma)	CGRP-levels were lower compared to healthy controls	Mean 20.75 years (1-55years)	High CGRP concentrations correlated with severe pruritus
Schmelz, 1997 [7]	Examine neuropeptide release in human skin elicited by histamine iontopheresis and topical CAP application	Experimental pain: histamine iontopheresis and CAP application	Samples from dermal microdialysis taken from 10 healthy volunteers. Patients were pain free prior start	Dermal microdialysis	CGRP concentration increased after histamine iontophoresis, but not capsaicin application	3 h	NR
Simone, 1998 [8]	Determine whether hyperalgesia after intradermal injection of CAP could be attributed to morphological changes in ENF’s	Experimental pain: intradermal CAP injection	Skin biopsies from 8 healthy volunteers	Tissue biopsies (skin)	Complete loss of CGRP-fibers was observed 72 h after capsaicin injections. They reappeared 3–4 weeks after	6 weeks	NR

*ACE* Angiotensin-converting enzyme, *AD* Atopic dermatitis, *CAP* Capsaicin injection, *ENF’s* Epidermal nerve fibers, *ES* Electrical current stimulation, *NEP* Neutral endopeptidase

Using the ELISA and dermal microdialysis method in healthy volunteers CGRP release was reported after electrical stimulation upon phosphoramidon but not after captopril infusion in the volar forearm [48]. Phosphoramidon and captopril, respectively, inhibit neutral endopeptidase and angiotensin-converting hormone, which are both involved in neuropeptide degradation [49].

Immunohistochemical analysis of skin biopsies in patients with painful scars from burn showed increased CGRP compared with controls with burn scars without pain [50]. Another study reported increased CGRP in hypertrophic burn scar compared to biopsies from unburned scars. Pain intensity was higher in patients with burn scars [51]. Moreover ELISA of peripheral blood showed increased CGRP levels up to 24 h after burn injuries compared with healthy volunteers [52] and in patients with pruritus due to atopic dermatitis [53]. Furthermore, CGRP levels were positively correlated with the severity of pruritus [53]. Nociceptive fibers have been shown to be involved in the sensation of pruritus [54].

### Neuropathic pain

We identified 11 studies in this category (Table 4). Radioimmunoassay showed higher serum CGRP levels in 19 patients with complex regional pain syndrome (CRPS) compared to controls. The difference was normalized after a 9-month pain management therapy [55]. In contrast another study found decreased serum CGRP levels in chronic CRPS patients (*n* = 12) compared with healthy controls [56]. No correlation between pain and CGRP levels was found in either study [55, 56]. Moreover, immunofluorescence analysis of skin biopsies from amputated limbs in CRPS patients showed loss of CGRP expression in two patients compared with skin biopsies from five controls. Correlation between pain measures and CGRP levels was not tested [57]. In post-herpetic neuralgia, increased CGRP expression in the affected skin compared with skin from a contralateral side in the same patient was reported by using immunofluorescence analyses of skin biopsies [58]. In one study using immunofluorescence of skin biopsies [59] no difference in CGRP expression was found between patients with chronic pain due to nerve injury after hand surgery and controls. The immunohistochemistry of peripheral nerve biopsies harvested from patients with Morton’s neuroma, which results in neuropathic pain, showed increased amount of CGRP in patients compared with controls [60].

**Table 4:** Studies on the role of CGRP in neuropathic pain

Study	Objectives	Reported pain as part of phenotype	Method and sample size	Source of CGRP	Results	Duration of the investigated condition	Correlation between CGRP level and pain
Albrecht, 2006 [1]	Investigate CGRP expression in skin from amputated extremity affected by CRPS	CRPS after amputation in upper and lower limbs	Skin tissue from 2 patients and 5 controls	Tissue biopsies (skin)	Loss of CGRP expression in CRPS patients	NR	NR
Attal, 2016 [2]	Determine CGRP levels in peripheral neuropathic pain patients after treatment with botulinum toxin A	Peripheral neuropathic pain, mixed group	ELISA of biopsy from 23 patients with active treatment and 17 patients with placebo treatment at week 1 and 4 after study start	Skin	No change in CGRP levels at week 4 compared to week 1. Average pain score was not changed either	NR	None
Awawdeh, 2002 [3]	Investigate presence of CGRP in the gingival crevicular fluid of teeth diagnosed with pain of pulpal origin	Tooth pain	Gingival crevicular fluid from a painful and non-painful site from 54 patients undergoing pulpectomy. 1 week after fluid was collected from 21 patients	Gingival crevicular fluid	No difference in CGRP-levels between painful tooth compared to the contralateral control tooth	NR	No clear association between CGRP-levels and dental pain
Boras, 2010 [4]	Determine saliva and serum CGRP levels in patients with BMS	Burning mouth syndrome	Saliva and serum from 26 patients and 22 controls	Saliva and serum	No difference between patients and controls	NR	NR
Birklein, 2001 [5]	Test contribution of neuropeptide release to pathophysiology of CRPS	CRPS in upper or lower limbs	Serum CGRP concentrations were measured in 19 patients on the affected (*n =* 19) side and non-affected side (*n =* 13) before and 9 months after therapy (*n =* 9). Comparison with controls (*n =* 16)	Blood (serum)	Increased CGRP levels in CRPS patients. No difference in CGRP levels in blood taken from affected versus non-affected side. After therapy – normalization of CGRP levels compared to healthy controls	Mean 29 weeks (range: 2 to 188 weeks)	No correlation between CGRP-levels and pain
Chavarría-Bolanos, 2014 [6]	Determine CGRP-levels in dental pulp tissue samples from 8 patients subjected to undergo controlled orthodontic intrusive forces	Tooth pain	Human premolar dental pulp tissue was extracted from 8 patients, and 8 controls	Tissue biopsies (dental pulp)	No differences in CGRP levels between the two groups	24 h	NR
Hou, 2011 [7]	Determine whether CGRP-IL is increased among epidermal keratinocytes in PHN and diabetes	PHN and small fiber neuropathy	Punch biopsies from 5 patients with PHN from painful areas, 5 patients with diabetes (biopsies from feet) and 11 controls	Tissue biopsies (skin)	Increased CGRP-levels in keratinocytes from PHN patients compared to controls	NR	CGRP levels were higher in painful skin areas compared to non-painful locations
Kalliomäki, 2011 [8]	Investigate structural and functional differences between patients with and without chronic pain following nerve injury	Hand pain due to nerve injury	Skin biopsies from 21 patients with pain and 9 controls without pain. All participants required hand surgery	Tissue biopsies (skin)	No difference in CGRP-staining between patients and controls	>1 year	No significant difference between pain and non-pain patients
Lindqvist. 2000 [9]	Examine CGRP expression in painful Morton’s neuroma patients	Forefoot Morton’s neuroma	11 nerve biopsies from 8 patients and 4 controls	Nerve biopsies	Increased levels of CGRP-IR nerve fibers compared to controls	NR	NR
Schinkel, 2009 [10]	Compare systemic inflammatory mediators in patients with CRPS I with controls	CRPS in the upper limb	Blood samples were obtained from 22 patients. 12 patients had chronic CRPS and 10 patients had acute CRPS. Patients were compared to 8 controls	Blood (plasma)	CGRP-levels were lower in chronic CRPS patients, compared to controls	Acute: < 6 months Chronic: > 6 months	NR
Zidverc-Trajkovic, 2009 [11]	Determine saliva CGRP levels in patients with BMS	Burning mouth syndrome	Saliva from 78 patients and 16 healthy controls	Saliva	Inconclusive, CGRP levels were non-significantly decreased in comparison to controls	At least 6 months	NR

*BMS* Burning mouth syndrome, *CRPS* Complex regional pain syndrome, *CSF* Cerebrospinal fluid

Four studies reported on CGRP radioimmunoassay: 1) gingival crevicular fluid in unilateral tooth pain patients [61] 2) saliva from burning mouth syndrome patients [62, 63], and 3) pulp biopsy in patients undergoing orthodontic intrusion [64]. None of the studies reported alteration in CGRP expressions in painful sides when compared with the non-painful side [61] or with controls [62, 64].

Attal et al. [65] investigated 152 patients with peripheral neuropathic pain of whom 68 were treated with botulinum toxin A and 66 received placebo. CGRP was analyzed in skin biopsies using ELISA at week 1 and 4 in 23 patients who received botulinum toxin A and in 17 patients who received placebo. No difference between groups was found [65].

### CGRP antagonists and antibodies and clinical trials

We did not identify any clinical trials on CGRP antagonists and antibodies for the treatment of non-headache pain by searching PubMed and Embase. Search on ClinicalTrial.gov for current CGRP antagonists and antibodies for the treatment of non-headache pain only yielded three studies.

The acute effect of PF-04427429 anti-CGRP monoclonal antibody, on attenuation of flare response after capsaicin challenge, used to induce experimental human pain, was studied in a double blind, randomized, placebo-controlled, third-party open, modified cross-over study in male healthy volunteers and using EMLA® cream as positive control [66]. However, primary outcome measure of the study was mean blood perfusion induced by capsaicin challenge (results not reported on ClinicalTrials.gov) and no pain perception measures were studied.

A phase 2 randomized, double-blind, placebo and active-controlled trial in patients with mild to moderate osteoarthritis knee pain failed to demonstrate efficacy of LY2951742, monoclonal antibody to CGRP [67]. The study was terminated. A total of 266 patients were randomized to 1 of 6 treatment arms: LY2951742 5 mg, 50 mg, 120 mg, or 300 mg, celecoxib 200 mg, or placebo. Using a Bayesian dose–response longitudinal model, response rates to all four LY2951742 treatment arms were not different from placebo while celecoxib met criteria for a positive study [68].

An ongoing study on remote ischemic conditioning in patients with ulcerative colitis a condition associated with abdominal pain and diarrhea is still in a recruiting phase [69]. Investigators plan to study changes of serum and mucosal CGRP levels (secondary endpoints) in patients with ischemic colitis after remote ischemic conditioning, a repeated brief and non-harmful suppression of blood circulation induced by placing a blood pressure cuff around the right or left arm.

## Discussion

### Summary of findings

The present review revealed the association between measured CGRP levels and somatic visceral, neuropathic and inflammatory pain. We found that in somatic pain conditions in particular, CGRP levels correlated with pain. Increased CGRP levels were reported in plasma, synovial and cerebrospinal fluid, tissue biopsies in individuals with degenerative disc disease, osteoarthritis and TMJ-pain. Furthermore, CGRP was elevated in acute pain conditions and pain after exercise.

In total 13 out of 20 studies on somatic pain increased levels of CGRP were reported. Five studies showed no difference or had no control group. Four out of eight studies investigated CGRP in experimental models of inflammatory pain. The remaining four studies reported elevated CGRP levels in patients with pain caused by scars and pruritus. There was no consensus regarding correlation between neuropathic pain and CGRP levels. Six out of eleven studies showed no difference in CGRP levels, three studies reported a positive correlation, and two studies reported a negative correlation between neuropathic pain and CGRP levels. In visceral pain conditions a correlation between gynecological pain and high CGRP levels were found in tissue biopsies and peritoneal fluid. However, only two studies used a control group or control conditions.

Thirty out of fifty studies (60%) included controls and suggested an association between CGRP levels and the respective pain condition. Twenty-six (52%) studies reported a positive association whereas four studies (8%) reported decreased CGRP levels. Studies reporting positive association investigated blood (10 studies), skin (5 studies), synovial tissue/fluid (5 studies), and other affected tissues (6 studies). Collectively, these studies showed a positive correlation between high CGRP levels and somatic pain conditions, especially osteoarthritis, acute muscular pain and chronic joint/muscular pain. These findings raise two important questions: what is the role of CGRP in the transmission of nociceptive signals and whether CGRP causes or modulates pain?

### CGRP and pathophysiology of pain

CGRP is widely distributed in the peripheral and central nervous system [70, 71] and CGRP receptors are expressed in pain pathways [72–76]. CGRP-like immunoreactivity (CGRP-LI) is found in 40–50% of dorsal root ganglia (DRG) neurons [77]. CGRP-LI was found C-fiber (46%), delta-fiber (33%), and A-alpha/beta fiber (17%) neurons [77]. Moreover, CGRP is usually co-localized with other neuropeptides, including substance P [78] and neurokinins [79] in DRG neurons. Peripheral CGRP-LI fibers terminate in lamina I, III and V of spinal cord [80] and CGRP-containing DRG neurons innervate joints [81]. Thus, CGRP and its receptors are widely distributed in peripheral and central pain pathways.

In animals CGRP can be released from peripheral and central nerve endings upon noxious pain mechanical stimulation of the skin [82–85]. In rats, the major part of circulating CGRP is released from perivascular nerve terminals [86, 87]. Acute and chronic nociception leads to altered release of CGRP from sensory nerve endings and central terminals into the dorsal horn of the spinal cord [88–91]. In rats, CGRP applied spinally causes facilitation of central excitability and central sensitization [92, 93]. Kessler et al. [94] demonstrated reduced mechanical allodynia in an animal model of OA following administration of an intrathecal CGRP receptor antagonist [94]. Animal in vitro studies reported direct activation of nociceptors by CGRP [95, 96]. CGRP injected into mouse hind paw skin produced mechanical allodynia [97]. In humans, however, a direct activation of nociceptive fibers is unlikely. CGRP injected intradermally or intramuscularly did not produce pain [98].

CGRP is also found in free nerve endings in skin and synovium) and perivascular afferents in different structures in both humans and animals [99–101]. The release of CGRP from these fibers causes vasodilation suggesting a role in neurogenic inflammation [98, 101, 102]. The question is whether CGRP exerts either pro- or anti-inflammatory/nociceptive effects. It is possible that CGRP release reflects the response of the nocifensor system to injury and inflammation to evoke protective vasodilatation. Deficiency of alpha CGRP (αCGRP knockout mice) was associated with enhanced inflammatory responses in the hippocampus and hypothalamus and reduced the survival rate compared to wild-type mice in septic shock condition [103]. However, αCGRP knockout mice displayed lower pain sensitivity to heat stimulation faster accumulation of c-Fos compared to wild-type animals after incision and complete Freund’s adjuvant injection [104]. In animals, sustained CGRP release may induce peripheral sensitization [105] likely due to release of inflammatory mediators (bradykinin, prostaglandins, etc.) from nerve endings and cells of immune system [106–108].

Inflammatory diseases of the joints tendons and discs may be associated with elevated levels of CGRP (Additional files 1 and 2: Tables S1 and S2). These data suggest that abnormal release of CGRP could be a marker of sensory afferent activation. Comparing CGRP changes in different tissue materials (i.e. blood, synovium, skin, CSF, ligament tissue, mucosa, etc.), it seems that elevated CGRP is more frequently found in blood, synovium and skin. Bullock et al. [109] suggested that CGRP release during joint degeneration in osteoarthritis might play an important role in the peripheral sensitization and proposed possible analgesic effect of CGRP antagonists in this condition. CGRP stimulates proliferation and migration of human endothelial cells [110], causing angiogenesis with the co-localized CGRP-containing perivascular nerve fibers. Intra-articular growth of CGRP-containing perivascular nociceptors have been reported in patients with osteoarthritis. It has further been shown that nociceptive nerve fibers innervating joints are sensitized in these patients [111] contributing to the experience of pain. Immunohistochemistry of forearm skin biopsies in patients with congenital insensitivity to pain (CIP) showed reduced amount of CGRP compared to controls [112]. Thus, measurement of CGRP may be regarded a marker of sensory afferent activation in the respective tissue during a pain condition [113]. This indicates that CGRP not only contributes to proliferation of CGRP-containing nociceptors, but could sensitize these nociceptors via neurogenic inflammation in humans. Whether CGRP causes pain *per se* can be examined by application of exogenous CGRP. Interestingly, dose-dependent angiogenesis after intra-articular CGRP injection in the rat knee can be blocked by the CGRP receptor antagonist, BIBN4096BS [114]. One way of exploring this hypothesis would be to study CGRP levels in humans after exposure to painful stimuli. In healthy volunteers, intradermal capsaicin injections produced a steady increase of CGRP levels in the first sampling period, but failed to reach significance in the second session [45]. The latter could be explained by capsaicin-induced desensitization of neuropeptide release from primary afferents [115]. Another study demonstrated that capsaicin-induced vasodilation in the human skin was mainly mediated by CGRP and not by other substances with vasodilator properties including prostaglandins, nitric oxide, or substance P [116]. Only few studies have investigated the effect of CGRP antagonist after intradermal capsaicin injections [66, 117]. Chi-Chung Li et al. [117] reported that CGRP antagonist MK-3207 inhibited capsaicin-induced vasodilation in skin. Sinclair et al. [118] demonstrated reduced increase in dermal blood flow after topical capsaicin application in the forearm of healthy volunteers who were pretreated with CGRP antagonist (telcagepant). The degree of inhibition in capsaicin-induced dermal blood flow was shown to be increased with higher LY2951742, CGRP monoclonal antibody, plasma concentrations suggesting dose–response relationship [119].

While increased CGRP levels in the affected tissue and synovial material indicate ingrowth of pain sensitive nerve fibers in the tissue it is unclear why CGRP level increases in blood and skin. CGRP is synthesized in central and peripheral neurons [120]. Two studies investigated CGRP levels in the cerebrospinal fluid during pain and found 1) no difference in cancer pain patients compared to controls [35], and 2) low CGRP levels in osteoarthrosis patients [25]. In contrast, biochemical studies in osteoarthrosis patients reported a positive association between pain and CGRP levels in blood [17, 18], synovial material [20–22], and skin [19]. Dermal electrical current stimulation in humans caused increased CGRP in blood [48]. However, a recent study randomized, double-blind, placebo and active-controlled study in patients with osteoarthritis knee pain did not demonstrate efficacy of LY2951742, monoclonal antibody to CGRP against placebo and the trial was terminated [68]. However, the study was only done in patients with mild and moderate symptoms. It is possible that patients with severe osteoarthritis involving other joints may respond differently. Other factors that may confound the results include the long duration of the disease (not reported in abstract), which can indicate presence of central sensitization and level of activity of patients that may worsen symptoms including pain. No studies to date have investigated the efficacy of monoclonal antibodies against CGRP *receptor* in patients with osteoarthritis knee pain.

Further studies addressing these issues are warranted.

## Conclusions

The present review suggests that CGRP may play a role in pain transmission in somatic pain conditions such as joint and muscular chronic pain. CGRP might have a pro-inflammatory role in peripheral nervous system by leading to release of pro-nociceptive substances and by facilitating central nociceptive transmission and contributing to central sensitization. However, the exact mechanisms and involvement of CGRP in nociceptive processing are not fully clarified. Understanding these mechanisms may lead to the potential development of new pharmacotherapies targeting CGRP and its receptors. Efficacy and safety of the CGRP antagonists and antibodies has already been established in migraine and this paves the way for more clinical trials in non-headache pain conditions.

## Additional files

Additional file 1: Table S1. Brief overview of used methods and the association between pain and CGRP in each category. (DOC 72 kb)
Additional file 2: Table S2. Brief overview of used methods and the association between pain and CGRP in the musculoskeletal category. (DOC 37 kb)

## Abbreviations

CGRP: Calcitonin gene-related peptide
CGRP-LI: Calcitonin gene-related peptide – like immunoreactivity
CIP: Congenital insensitivity to pain
CNS: Central nervous system
CRPS: Complex regional pain syndrome
DRG: Dorsal root ganglia
TMJ: Temporomandibular joint
