# Supplementary material for: Calcitonin gene-related peptide and pain: a systematic review
Source: J Headache Pain. 2017 Mar 16;18(1):34. doi: 10.1186/s10194-017-0741-2 (PMC5355411; doi:10.1186/s10194-017-0741-2)
Supplement: Additional file 1: Table S1. — Brief overview of used methods and the association between pain and CGRP in each category. (DOC 72 kb) [file 10194_2017_741_MOESM1_ESM.doc]

**Supplementary table 1**

| **Category** | **Condition** | **Study** | **Methods** | **Material** | **Pain-CGRP association** |
| --- | --- | --- | --- | --- | --- |
| Inflammatory | Experimental | Schmelz, 1997 | ELISA | Blood | NR |
| Inflammatory | Experimental | Geber, 2007 | ELISA | Blood | None |
| Inflammatory | Experimental | Krämer, 2005 | ELISA | Blood | Positive |
| Inflammatory | Experimental | Simone, 1998 | IHC | Skin | NR |
| Inflammatory | Pruritus | Salomon, 2008 | ELISA | Blood | Positive |
| Inflammatory | Scars | Onuoha, 2001 | ELISA | Blood | Positive |
| Inflammatory | Scars | Hamed, 2011 | IHC | Skin | Positive |
| Inflammatory | Scars | Kwak, 2014 | IHC | Skin | Positive |
| Neuropathic | Mixed group | Attal, 2016 | ELISA | Skin | None |
| Neuropathic | CRPS | Schinkel, 2009 | RIA | Blood | Negative |
| Neuropathic | CRPS | Birklein, 2011 | RIA | Blood | Positive |
| Neuropathic | CRPS | Albrecht, 2006 | IF | Skin | Negative |
| Neuropathic | MN | Lindqvist, 2000 | IHC | Peripheral nerve | Positive |
| Neuropathic | Oral pain | Awawdeh, 2002 | RIA | Gingival crevicular fluid | None |
| Neuropathic | Oral pain | Boras, 2010 | RIA | Saliva and serum | None |
| Neuropathic | Oral pain | Chavarria-Bolanos, 2014 | RIA | Pulp | None |
| Neuropathic | Oral pain | Zidverc-Trajkovic, 2009 | RIA | Saliva | None |
| Neuropathic | PNH | Hou, 2011 | IF | Skin | Positive |
| Neuropathic | Postsurgical | Kalliomäki, 2011 | IF | Skin | None |
| Somatic | Cancer | Samuelsson, 1993 | RIA | CSF | None |
| Somatic | DDD | Ozawa, 2006 | IHC | Disc | No control group |
| Somatic | DDD | Brown, 1997 | IHC | Disc | Positive |
| Somatic | Exercise | Jonhagen, 2006 | RIA | Blood | Positive |
| Somatic | Injury | Alpar, 2002 | ELISA | Blood | Positive |
| Somatic | Injury | Takeuchi, 2007 | ELISA | Blood | Positive |
| Somatic | Injury | Onuoha, 1999 | ELISA | Blood | Positive |
| Somatic | Injury | Larsson, 1991 | RIA | Synovium | Positive |
| Somatic | OA | Dong, 2015 | ELISA | Blood | Positive |
| Somatic | OA | Lin, 2015 | ELISA | Blood | Positive |
| Somatic | OA | Lindh, 1999 | RIA | CSF | Negative |
| Somatic | OA | Ikeuchi, 2012 | IHC | Ligament/tendon | None |
| Somatic | OA | Carlsson, 2006 | IF | Skin | Positive |
| Somatic | OA | Saxler, 2007 | IHC | Synovium | Positive |
| Somatic | OA | Wang, 2015 | IHC | Synovium | Positive |
| Somatic | OA | Takeshita, 2012 | IHC | Synovium | Positive |
| Somatic | Tendinopathy | Sasaki, 2013 | IHC | Ligament/tendon | Negative |
| Somatic | Tendinopathy | Danielson, 2008 | IHC | Ligament/tendon | No control group |
| Somatic | Tendinopathy | Bjur, 2005 | IHC | Ligament/tendon | None |
| Somatic | TMJ | Sato, 2004 | IHC | Synovium | Positive |
| Visceral | Cardiac | Währborg, 1999 | RIA | Blood | None |
| Visceral | Gastrointestinal | Yoshida, 2013 | ELISA | Gastric mucosa | None |
| Visceral | Gastrointestinal | Mönnikes, 2005 | RIA | Gastric mucosa | None |
| Visceral | Gastrointestinal | Büchler, 1992 | IHC | Pancreatic tissue | Positive |
| Visceral | Gynecological | Tokushige, 2007 | IHC | Endo-/myometrium tissue | Positive |
| Visceral | Gynecological | Tokushige, 2006 | IHC | Endometriotic lesions | Positive |
| Visceral | Gynecological | Barcena de Arellano, 2011 | IF | Peritoneal fluid | Positive |
| Visceral | Gynecological | Tympanidis, 2003 | IHC | vulval tissue | None |
