# Supplementary material for: Calcitonin gene-related peptide and pain: a systematic review
Source: J Headache Pain. 2017 Mar 16;18(1):34. doi: 10.1186/s10194-017-0741-2 (PMC5355411; doi:10.1186/s10194-017-0741-2)
Supplement: Additional file 2: Table S2. — Brief overview of used methods and the association between pain and CGRP in the musculoskeletal category. (DOC 37 kb) [file 10194_2017_741_MOESM2_ESM.doc]

**Supplementary table 2**

| **Condition** | **Study** | **Methods** | **Material** | **Pain-CGRP association** |
| --- | --- | --- | --- | --- |
| DDD | Brown, 1997 | IHC | Disc | Positive |
| Injury | Alpar, 2002 | ELISA | Blood | Positive |
| Injury | Takeuchi, 2007 | ELISA | Blood | Positive |
| Injury | Onuoha, 1999 | ELISA | Blood | Positive |
| Injury | Larsson, 1991 | RIA | Synovium | Positive |
| OA | Dong, 2015 | ELISA | Blood | Positive |
| OA | Lin, 2015 | ELISA | Blood | Positive |
| OA | Carlsson, 2006 | IF | Skin | Positive |
| OA | Saxler, 2007 | IHC | Synovium | Positive |
| OA | Wang, 2015 | IHC | Synovium | Positive |
| OA | Takeshita, 2012 | IHC | Synovium | Positive |
| Tendinopathy | Sasaki, 2013 | IHC | Ligament/tendon | Negative |
| OA | Lindh, 1999 | RIA | CSF | Negative |
| Tendinopathy | Bjur, 2005 | IHC | Ligament/tendon | None |
| OA | Ikeuchi, 2012 | IHC | Ligament/tendon | None |
| Tendinopathy | Danielson, 2008 | IHC | Ligament/tendon | No control group |
| DDD | Ozawa, 2006 | IHC | Disc | No control group |
